# TLR ligand induced IL-6 counter-regulates the anti-viral CD8^**+**^ T cell response during an acute retrovirus infection

**DOI:** 10.1038/srep10501

**Published:** 2015-05-21

**Authors:** Weimin Wu, Kirsten K. Dietze, Kathrin Gibbert, Karl S. Lang, Mirko Trilling, Huimin Yan, Jun Wu, Dongliang Yang, Mengji Lu, Michael Roggendorf, Ulf Dittmer, Jia Liu

**Affiliations:** 1 Institute for Virology, University Hospital of Essen, University of Duisburg-Essen, Essen, Germany; 2 Institute for Immunology, University Hospital of Essen, University of Duisburg-Essen, Essen, Germany; 3 Wuhan Institute of Virology, Chinese Academy of Sciences, Wuhan, China; 4 Department of Infectious Diseases, Union Hospital, Tongji Medical College, Huazhong University of Science and Technology, Wuhan, China

## Abstract

We have previously shown that Toll-like receptor (TLR) agonists contribute to the control of viral infection by augmenting virus-specific CD8^+^ T-cell responses. It is also well established that signaling by TLRs results in the production of pro-inflammatory cytokines such as interleukin 6 (IL-6). However, how these pro-inflammatory cytokines influence the virus-specific CD8^+^ T-cell response during the TLR agonist stimulation remained largely unknown. Here, we investigated the role of TLR-induced IL-6 in shaping virus-specific CD8^+^ T-cell responses in the Friend retrovirus (FV) mouse model. We show that the TLR agonist induced IL-6 counter-regulates effector CD8^+^ T-cell responses. IL-6 potently inhibited activation and cytokine production of CD8^+^ T cells *in vitro*. This effect was mediated by a direct stimulation of CD8^+^ T cells by IL-6, which induced upregulation of STAT3 phosphorylation and SOCS3 and downregulated STAT4 phosphorylation and T-bet. Moreover, combining TLR stimulation and IL-6 blockade during an acute FV infection resulted in enhanced virus-specific CD8^+^ T-cell immunity and better control of viral replication. These results have implications for our understanding of the role of TLR induced pro-inflammatory cytokines in regulating effector T cell responses and for the development of therapeutic strategies to overcome T cell dysfunction in chronic viral infections.

Toll-like receptors (TLRs) play a crucial role in early host defense by recognizing exogenous ligands associated with pathogenic microorganisms - “pathogen-associated molecular patterns” (PAMPs). Recognizing PAMPs and activating signaling pathways by TLRs on various immune cells launch immediate tissue specific and global responses of the innate immune system to the invading pathogens. Besides, recent advances in understanding the nature and functions of TLRs revealed the importance of these receptors for the extensive cross-talk between innate and adaptive immunity^1^. TLRs have been shown to play an important role in the initiation and modulation of adaptive immune responses, T cell differentiation, and immune tolerance^2^. For example, ligation of TLRs causes functional maturation of professional antigen-presenting cells (APCs), in particular dendritic cells (DCs)^3^. This leads to increased expression of costimulatory molecules such as CD80/CD86 and increased release of cytokines such as interleukin (IL)-12, which are both required to promote cytotoxic CD8^+^ T cell differentiation and activation^4^,^5^,^6^. We recently showed that TLR can switch liver APC from a tolerogenic to an immunogenic state by triggering cytokine production of APCs and promote the development of T cell immunity^7^.

In response to TLR stimulation, both lymphoid and non-lymphoid cells can produce proinflammatory cytokines. Together with TNF-α and IL-1, IL-6 is considered to be the major proinflammatory cytokine induced by TLR agonists^8^. This multifunctional cytokine has been demonstrated to be important for initiating innate immunity, and regulating adaptive immune responses^9^ and thus is involved in the protection from pathogens during an infection^10^,^11^,^12^. IL-6, originally identified as B cell stimulating factor-2, induces the differentiation of activated B cells into Immunoglobulin (Ig)-producing plasma cells^13^. IL-6 is also an important modulator of CD4^+^ T cell effector functions thereby shaping the cellular immune response^14^,^15^,^16^,^17^. IL-6 in combination with transforming growth factor (TGF)-β preferentially induces the differentiation of naïve CD4^+^ T cells into Th17 cells^18^, and inhibits TGF-β induced regulatory T cell (Treg) development^19^. The IL-6–induced dominance of Th17 cells over Tregs may be responsible for overcoming immunological tolerance and the development of inflammatory autoimmune diseases^20^. Therefore, IL-6 is considered one of the key factors that contribute to the modulation of adaptive immune response after TLR ligand stimulation.

However, despite numerous preclinical and clinical studies that aim to develop TLR agonists into drugs for the treatment of infectious diseases, the effect of TLR induced IL-6 on virus-specific CD8^+^ T cell responses remains largely unknown. In this study, we determined whether and how IL-6 blockade during TLR ligand stimulation regulates virus-specific CD8^+^ T cell responses in the Friend retrovirus (FV) mouse model. FV infection model represents a well established animal model to study T cell immunity to retroviruses^21^. For a therapeutic purpose, TLR ligands have been used in the FV model to augment virus-specific immune responses and improve control of virus replication^22^,^23^. Here, we show that TLR-induced IL-6 production counter-regulates the differentiation of FV-specific CD8^+^ T cells into effector T cells. Compared to TLR ligand treatment alone, additional IL-6 blockade results in better generation of virus-specific CD8^+^ T cell immunity and improved control of virus replication.

## Results

### TLR-induced IL-6 inhibits effector CD8^+^ T cell responses

**Figure 1:**
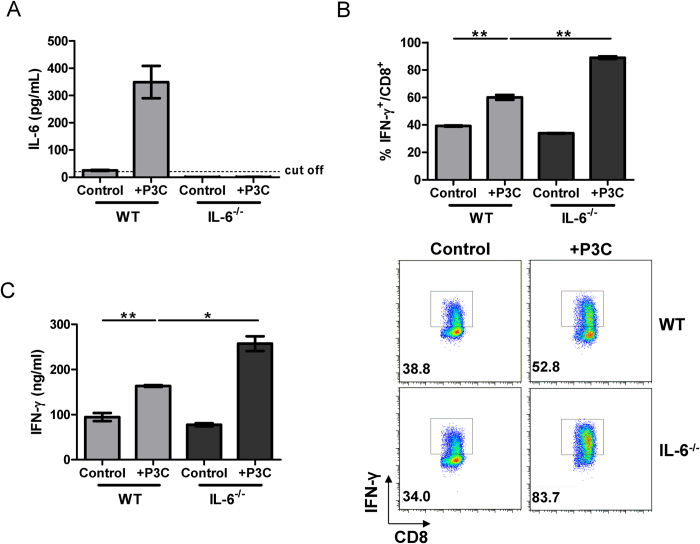
Effect of TLR-induced IL-6 on effector CD8^+^ **T cell responses.** Total splenocytes from C57BL/6 wild type (WT) or IL-6 knockout (IL-6−/−) mice were stimulated with anti-CD3 (1 μg/ml) and anti-CD28 (1 μg/ml), P3C (1 μg/ml) was added or not as indicated. (***A**)* Supernatant was collected after 48 h and IL-6 production was determined by ELISA. *(**B**)* The IFN-γ production by CD8^+^ T cells was measured by intracellular cytokine staining after 72 h. Representative dot plots show the population of IFN-γ^+^ CD8^+^ T cells (lower panel). *(**C**)* The IFN-γ levels in the supernatant were measured by ELISA after 72 h. Sample of each treatment was measured in duplicates for ELSIA or FACS. Data shown are the mean ± SD of one representative experiment out of three independent experiments. *p < 0.05; **p < 0.01, ***p < 0.001.

To confirm IL-6 production by splenocytes after TLR stimulation, total splenocytes from WT or IL-6 knockout (KO) mice were activated by αCD3/αCD28 antibodies and additionally stimulated with the TLR1/2 agonist P3C. The IL-6 concentration in the cell culture supernatant was determined by ELISA. As expected, P3C stimulation induced strong IL-6 production by splenocytes from WT mice. The IL-6 was barely detectable in WT splenocytes that did not receive P3C and was undetectable in IL-6^−/−^ splenocytes with or without P3C stimulation (Fig. 1A). To examine the contribution of the αCD3/αCD28 activation and the P3C stimulation to the overall IL-6 production, we compared the P3C induced IL-6 levels of non-activated splenocytes and αCD3/αCD28 activated splenocytes. P3C stimulation alone induced less IL-6 production in splenocytes (304.3 pg/ml in average) than the combination of P3C and αCD3/αCD28 stimulation (453.4 pg/ml in average) (Supplemental Fig. 1). This result indicates that about one third of the IL-6 produced after costimulation with αCD3/αCD28 and P3C came from the activated T cells. Next, we investigated whether the absence of TLR-induced IL-6 would influence effector CD8^+^ T cell differentiation. In response to αCD3/αCD28 activation, which induced only marginal amounts of IL-6, WT and IL-6^−/−^ splenocytes showed no significant difference in the percentage of interferon (IFN)-γ producing CD8^+^ T cells. As expected, additional P3C stimulation significantly enhanced the frequency of IFN-γ producing effector CD8^+^ T cells in WT splenocytes, and this effect was surprisingly even more pronounced in splenocytes from IL-6 KO mice (Fig. 1B). Measuring secreted IFN-γ in the supernatant of the cell cultures by ELISA confirmed that the highest production of IFN-γ was found in P3C-stimulated splenocytes that were deficient for IL-6 (Fig. 1C).

### Exogenous IL-6 inhibits effector CD8^+^ T cell responses

**Figure 2:**
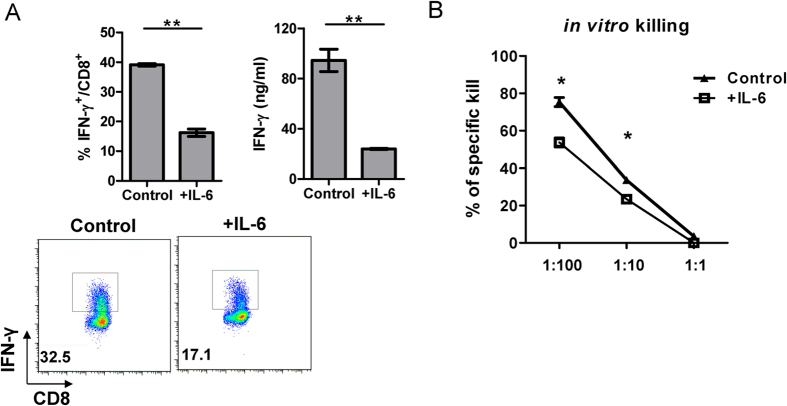
Effect of exogenous IL-6 on effector CD8^+^ **T cell responses.** *(**A**)* Total splenocytes from C57BL/6 wild type mice were stimulated with anti-CD3 (1 μg/ml) and anti-CD28 (1 μg/ml), IL-6 (10 ng/ml) was added or not as indicated. The IFN-γ production by CD8^+^ T cells was measured by intracellular cytokine staining (left panel) and ELISA (right panel) after 72 h. Representative dot plots show the population of IFN-γ^+^ CD8^+^ T cells (lower panel). *(**B**)* Total splenocytes from FV-TCR TCR transgenic mice were stimulated with 2 μg/ml FV peptide (FV GagL CTL epitope aa 85–93) for 3 days, IL-6 (10 ng/ml) was added or not as indicated. Activated FV-TCR TCR transgenic CD8+ T cells were analyzed for their cytotoxic potential against FV peptide-loaded target cells in an *in vitro* kill assay. Sample of each treatment was measured in duplicates for ELSIA or FACS. Data shown are the mean ± SD of one representative experiment out of three independent experiments. ^*^p < 0.05; ^**^p < 0.01, ^***^p < 0.001.

Our results indicated that TLR ligand-induced IL-6 negatively regulates effector CD8^+^ T cell responses. To confirm this effect in a TLR ligand independent system, exogenous IL-6 was added to αCD3/αCD28 activated splenocytes from WT mice and the percentage of IFN-γ producing CD8^+^ T cells was measured. As expected, significantly less IFN-γ producing CD8^+^ T cells and lower levels of IFN-γ in the supernatant were detected when exogenous IL-6 was added (Fig. 2A). Next, we investigated whether exogenous IL-6 influences the IFN-γ production by CD8^+^ T cells from WT and IL-6 KO mice treated with P3C. Adding IL-6 to P3C-stimulated WT splenocytes only slightly decreased the IFN-γ production by CD8^+^ T cells, indicating that P3C stimulation already induced saturated amounts of IL-6 that regulate CD8^+^ T cell functions. However, adding exogenous IL-6 to P3C stimulated IL-6 KO splenocytes resulted in a significant decrease in IFN-γ production by CD8^+^ T cells and fully abolished the promoting effect of IL-6 deficiency on the effector CD8^+^ T cell activation, as P3C stimulated WT splenocytes and IL-6 KO splenocytes showed comparable levels of IFN-γ production in the presence of exogenous IL-6 (Supplemental Fig. 2). This result further proved that IL-6 negatively regulates effector CD8^+^ T cell responses after polyclonal T cell activation.

To examine whether IL-6 also regulates the cytotoxic activity of effector CD8+ T cells, we performed an *in vitro* killing assay utilizing T-cell receptor (TCR) transgenic CD8+ T cells specific for the DbGagL FV epitope (FV-TCR CD8+ T cells). Naive splenocytes from FV-TCR transgenic mice were stimulated with DbGagL FV peptide to induce effector T cell differentiation, and exogenous IL-6 was added or not. The ability of differently treated effector CD8+ T cells to kill epitope peptide-loaded target cells was compared. As expected, IL-6 treatment led to a reduced cytotoxic activity of effector CD8+ T cells, as they were less efficient in killing peptide-loaded target cells *in vitro* than cells that did not receive IL-6 (Fig. 2B). Taken together, these results indicate that IL-6 inhibits several effector CD8+ T cell responses.

### IL-6 directly inhibits effector CD8^+^ T cell differentiation through the STAT3 signaling pathway

**Figure 3:**
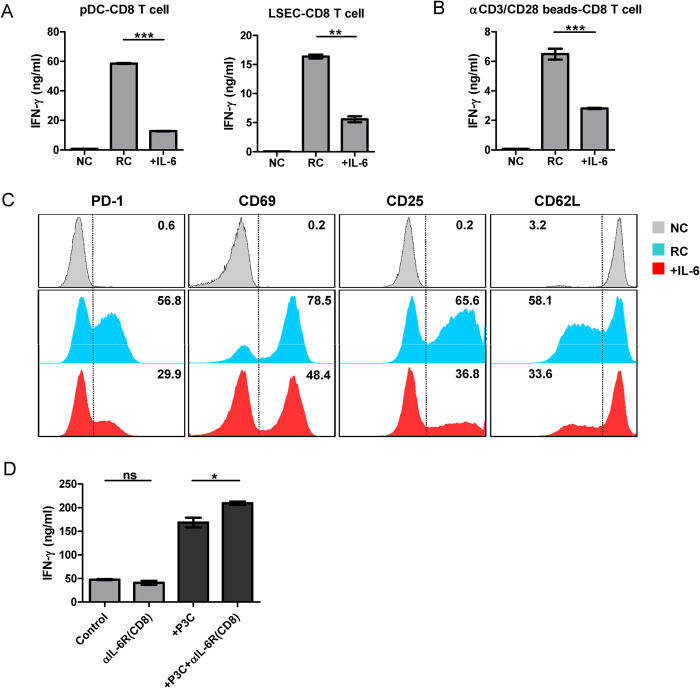
Direct effect of IL-6 on effector CD8^+^ **T cell differentiation.** *(**A**)* Splenic DCs or LSECs were pulsed with 2 μg/ml FV peptide (FV GagL CTL epitope aa 85–93) and cocultured with purified FV-TCR TCR transgenic CD8^+^ T cells in the presence of 10 ng/ml IL-12 (RC: responder control). DCs or LSECs without peptide loading served as a negative control (NC). IL-6 (10 ng/ml) was added or not as indicated. The IFN-γ production by CD8^+^ T cells was measured by ELISA after 72 h. (***B**)* Purified CD8^+^ T cells from C57BL/6 wild type mice were cocultured with CD3/CD28 Dynabeads in the presence of 10 ng/ml IL-12 (RC: responder control), unstimulated CD8^+^ T cells served as a negative control (NC). IL-6 (10 ng/ml) was added or not as indicated. The IFN-γ production by CD8^+^ T cells was measured by ELISA after 72 h. *(**C**)* CD8^+^ T cells treated as in panel B were measured for effector T cell marker expression (PD-1, CD69, CD25, CD62L) by flow cytometry. The numbers in the panel indicate the percentage of PD-1^+^, CD69^+^, CD25^+^, and CD62L^−^ cells. *(**D**)* CD8^+^ T cells were purified from total splenocytes and incubated with anti-IL-6R antibody (10 μg/ml) or not for 1 h. The control or IL-6R blocked CD8^+^ T cells were then mixed with other splenocytes and were stimulated with anti-CD3 (1 μg/ml) and anti-CD28 (1 μg/ml). P3C (1 μg/ml) was added or not as indicated. The IFN-γ production by CD8^+^ T cells was measured by ELISA after 48 h. Sample of each treatment was measured in duplicates for ELSIA or FACS.

Next, we examined the mechanism of IL-6 mediated effector CD8^+^ T cell regulation. Firstly, we asked whether IL-6 acts on APCs or directly on CD8^+^ T cells to regulate effector CD8^+^ T cell differentiation. To answer this question, an antigen-specific CD8^+^ T cell activation assay was established. Purified naive FV-specific TCR transgenic CD8^+^ T cells specific for the DbGagL FV epitope were primed with their cognate antigen peptide loaded onto APCs of different origin (splenic DCs or liver sinusoidal endothelial cells). Liver sinusoidal endothelial cells (LSECs) are unique liver-resident APCs capable of antigen cross-presentation and subsequent tolerization of naïve CD8^+^ T cells. However, in the presence of IL-12, peptide presentation by both APCs results in activation and IFN-γ production by the TCR transgenic CD8^+^ T cells^7^. Similar to our previous results, exogenous IL-6 potently inhibited the cytokine production independent of the APC type that was used for peptide presentation (Fig. 3A). This result implies that IL-6 may directly act on CD8^+^ T cells to modulate their function. To confirm this conclusion, highly purified CD8^+^ T cells (over 99%) were activated by CD3/CD28 Dynabeads and IL-12. This resulted in potent IFN-γ production, which was significantly decreased when exogenous IL-6 was added (Fig. 3B). IL-6 also changed the phenotype of the activated CD8^+^ T cells, as IL-6 treatment reduced the expression of the activation markers PD-1, CD69 and CD25, and increased CD62L expression compared to T cells cocultured with only Dynabeads and IL-12 (Fig. 3C). To confirm that IL-6 is directly effecting CD8^+^ T cell responses, we selectively blocked IL-6R on CD8^+^ T cells by sorting CD8^+^ T cells from total splenocytes and incubating them with an IL-6R blocking antibody. The IL-6R blocked CD8^+^ T cells were then mixed with non-treated splenocytes and were stimulated with αCD3/αCD28 antibodies. P3C was added or not as indicated. P3C stimulation significantly enhanced the IFN-γ production by CD8^+^ T cells. Importantly, combining P3C stimulation and selective blocking of IL-6R on CD8^+^ T cells led to a further enhancement of the IFN-γ production by CD8^+^ T cells (Fig. 3D). These results show that IL-6 can directly act on CD8^+^ T cells and negatively regulates their effector function.

**Figure 4:**
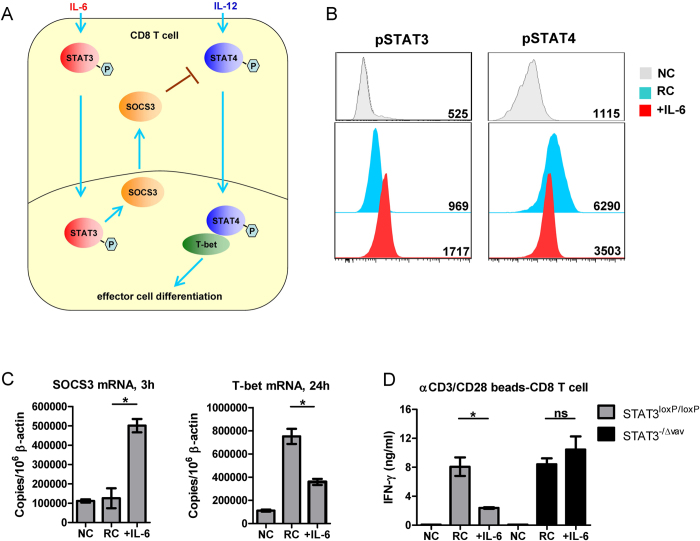
Mechanism of IL-6 mediated inhibition of effector CD8^+^ **T cell differentiation.** *(**A**)* Mechanism schema of the IL-6 mediated inhibition of effector CD8^+^ T cell differentiation. *(**B**)* Purified CD8^+^ T cells from C57BL/6 wild type mice were cocultured with CD3/CD28 Dynabeads in the presence of 10 ng/ml IL-12 (RC: responder control), unstimulated CD8^+^ T cells served as a negative control (NC). IL-6 (10 ng/ml) was added or not as indicated. The phosphorylation status of STAT3 and STAT4 of CD8^+^ T cells by flow cytometry after 1 h. The numbers in the panel indicate the mean florescence intensity of pSTAT-3 and pSTAT-4. *(**C**)* CD8^+^ T cells treated as in panel B were measured for SOCS3 and T-bet transcriptional expression by real-time RT-PCR at indicated time points. *(**D**)* Purified CD8^+^ T cells from STAT3^loxP/loxP^ or STAT3^−/∆vav^ mice were cocultured with CD3/CD28 Dynabeads in the presence of 10 ng/ml IL-12 (RC: responder control). IL-6 (10 ng/ml) was added or not as indicated. The IFN-γ production by CD8^+^ T cells was measured by ELISA after 72 h. Sample of each treatment was measured in duplicates for ELSIA or FACS. Data shown are the mean ± SD of one representative experiment out of three independent experiments. ^*^p < 0.05; ^**^p < 0.01, ^***^p < 0.001.

Next, we examined the molecular mechanism of the IL-6 mediated regulation of CD8^+^ T cells activation. Since it is well known that binding of IL-6 to its receptor initiates phosphorylation and activation of signal transducer and activator of transcription (STAT) 3, we hypothesized that direct IL-6 stimulation of CD8^+^ T cells activates STAT3 and subsequently upregulates expression of suppressor of cytokine signaling (SOCS) 3^24^. SOCS3 can dampen IL-12-dependent phosphorylation of STAT4 signaling which is critical for T-bet expression and effector CD8^+^ T cell differentiation^24^ (Fig. 4A). To test this hypothesis, phosphorylation of STAT3 (pSTAT3) and STAT4 (pSTAT4) as well as the expression of SOCS3 and T-bet were examined in activated CD8^+^ T cells after IL-6 treatment. Control CD8^+^ T cells isolated from splenocytes were activated with Dynabeads and IL-12, which induced expression of pSTAT4 and of T-bet mRNA but only minimal expression of SOCS3 mRNA (Fig. 4B,C). Additional IL-6 treatment shifted the expression from pSTAT4 to pSTAT3 and significantly upregulated SOCS3 mRNA levels correlating with reduced T-bet mRNA levels (Fig. 4B,C). A previous study has shown that IL-6 can also upregulate the expression of SOCS1 in CD4 T cells, which inhibits IFN-γ signaling and Th1 differentiation^17^. Therefore, we also examined SOCS1 expression in CD8^+^ T cells after IL-6 treatment. Compared to activated control CD8^+^ T cells, IL-6 treatment did not significantly upregulate SOCS1 mRNA levels (Supplemental Fig. 3) suggesting that this inhibitor does not play a role in IL-6 mediated T cell suppression. To further investigate the role of STAT3 pathway in the IL-6 mediated CD8^+^ T cell regulation, we employed STAT3^−/∆vav^ mice in which STAT3 deficiency is restricted to the hematopoietic compartment. Littermate STAT3^loxP/loxP^ mice with normal STAT3 expression were used as controls. Splenic CD8^+^ T cells were purified from STAT3^loxP/loxP^ or STAT3^−/∆vav^ mice and were activated by αCD3/CD28 Dynabeads and IL-12. Consistent with previous result, exogenous IL-6 potently inhibited the IFN-γ production of activated STAT3^loxP/loxP^ CD8^+^ T cells. In contrast, no significant decrease of IFN-γ produced by STAT3^−/∆vav^ CD8^+^ T cells was observed when exogenous IL-6 was added, showing that STAT3 deficiency totally abolished the inhibitory effect of IL-6 on CD8^+^ T cell function (Fig. 4D).

Another possible mechanism for IL-6 counter-regulation of CD8^+^ T cell responses is that IL-6 may interfere with the TCR signaling pathway. Since TCR ligation-induced gene expression and T cell activation rely on an early burst of protein-tyrosine kinase-induced phosphorylation, we examined whether IL-6 stimulation changes the tyrosine phosphorylation pattern of TCR- and CD28-stimulated CD8^+^ T cells. Purified naive CD8^+^ T cells were stimulated with CD3/CD28 Dynabeads, and exogenous IL-6 was added or not. At the indicated time points, cells were harvested for tyrosine phosphorylation analysis. Compared to non-stimulated control cells, TCR and CD28 stimulated CD8+ T cells showed a different tyrosine phosphorylation pattern. However, exogenous IL-6 stimulation did not change the tyrosine phosphorylation patterns of TCR-stimulated CD8^+^ T cells at any of the investigated time points (Supplemental Fig. 4). Therefore, IL-6 does most likely not interfere with the TCR signaling pathway.

Taken together, these results indicate that IL-6 directly inhibits effector CD8^+^ T cell activity by inducing the STAT3 signaling pathway and suppressing the STAT4 signaling pathway.

### IL-6 treatment inhibits effector CD8^+^ T cell responses in acute FV infected mice

**Figure 5:**
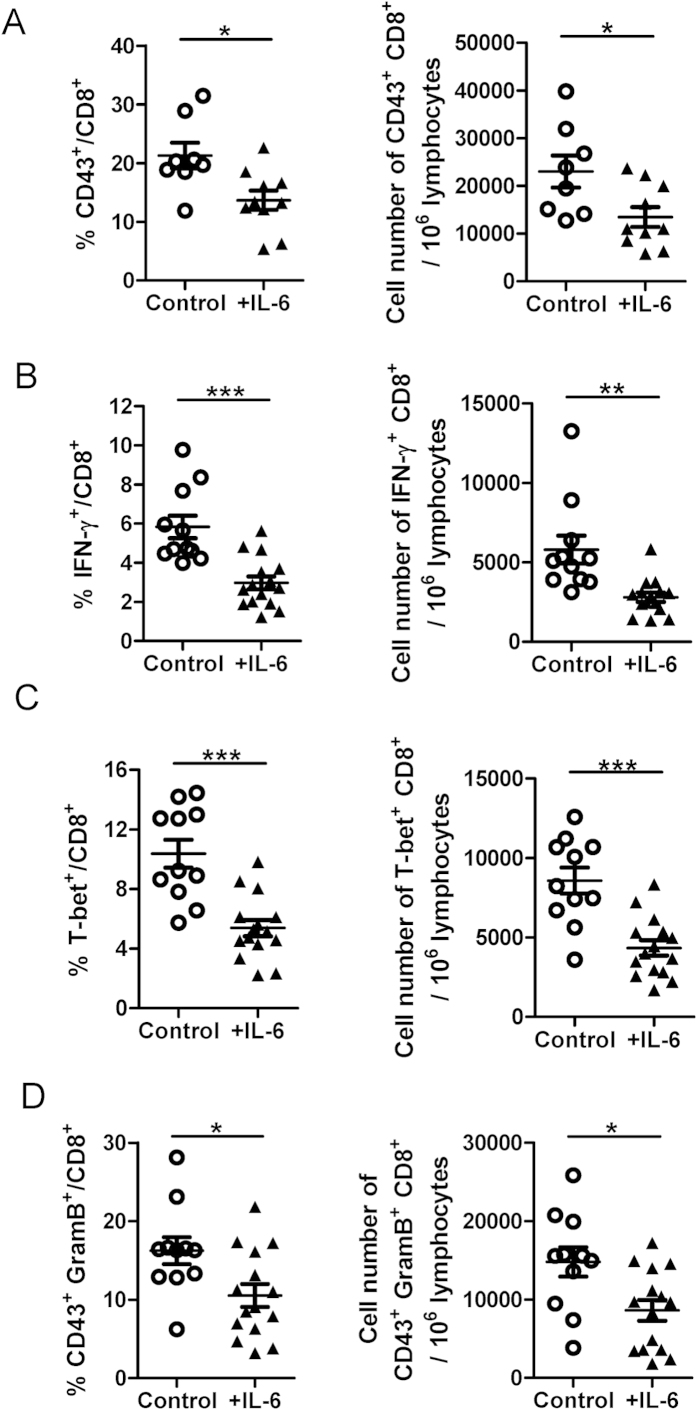
Effect of *in vivo* IL-6 administration on effector CD8^+^ **T cell differentiation in acute FV infection.** From 5 dpi to day 9 dpi, acute FV infected mice were treated with IL-6 (2 μg i.p.) daily or not. Mice were sacrificed on 10 dpi. *(**A**)* CD43 expression in splenic CD8^+^ T cells was measured by flow cytometry (Control: n = 8, +IL-6: n = 10). *(**B**)* IFN-γ production by splenic CD8^+^ T cells was measured by intracellular cytokine staining (Control: n = 11, +IL-6: n = 15). *(**C**)* T-bet expression in splenic CD8^+^ T cells was measured by flow cytometry (Control: n = 11, +IL-6: n = 15). *(**D**)* The expression of granzyme B in CD43^+^ CD8 T cells was measured by intracellular staining (Control: n = 11, +IL-6: n = 15). ^*^p < 0.05; ^**^p < 0.01, ^***^p < 0.001.

An important question is whether the suppressive effect of IL-6 on effector CD8^+^ T cells can also be demonstrated *in vivo*. To answer this question, we explored the FV mouse model, which represents a well-established small animal model to study T cell immunity to retroviruses^21^. C57BL/6 mice were infected with FV and treated daily with IL-6 during the induction phase of effector T cells in FV infection (5 dpi to 9 dpi). On day 10 pi effector CD8^+^ T cell functions were analyzed. Compared to controls, mice that received IL-6 treatment showed significantly reduced percentages and numbers of activated (CD43^+^) CD8^+^ T cells in the spleen (Fig. 5A). More importantly, after *ex vivo* restimulation, significantly fewer splenic CD8^+^ T cells of the IL-6 treated mice produced IFN-γ (Fig. 5B). Similar to our *in vitro* data, IL-6 also significantly downregulated T-bet expression in CD8^+^ T cells during FV infection (Fig. 5C). To analyze the cytolytic effector function of activated CD8^+^ T cells during the IL-6 treatment, we measured intracellular expression of the cytotoxic molecule granzyme B in activated (CD43^+^) CD8^+^ T cells. IL-6 treated mice had a 50% decrease in both the percentage and absolute numbers of granzyme B-producing CD43^+^ CD8^+^ T cells compared to non-treated control mice (Fig. 5D). These results show that IL-6 treatment can interfere with the effector functions of CD8^+^ T cells during acute FV infection.

### Combining TLR stimulation and IL-6 blockade results in potent anti-viral T cell immunity and control of virus replication in FV infection

Our results indicate that TLR-induced IL-6 counter-regulates the promoting effects of TLR ligands on virus-specific CD8^+^ T cell immunity. Thus, combining TLR ligand treatment and IL-6 blockade may result in augmented CD8^+^ T cell immunity and virus control compared to TLR ligand therapy alone.

**Figure 6:**
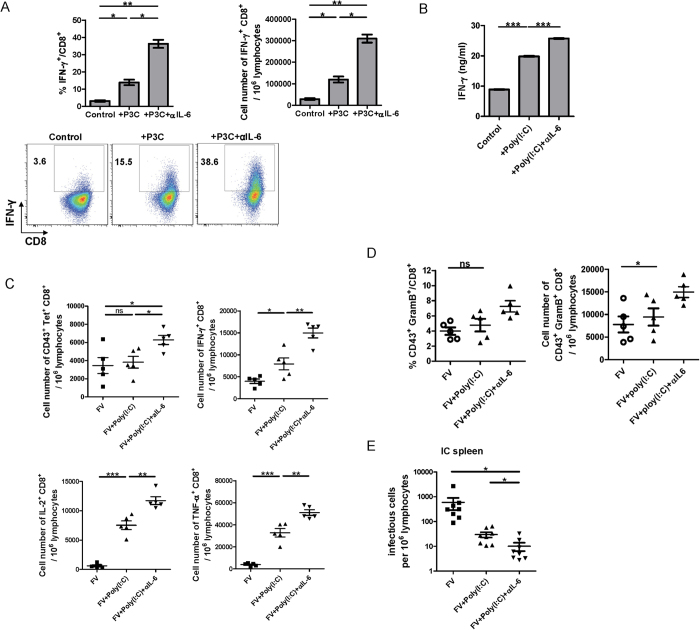
Analysis of combining TLR stimulation and IL-6 blockade on triggering virus-specific CD8^+^ T cell immunity *in vitro and in vivo*. *(**A**)* Total splenocytes from FV-TCR TCR transgenic mice were stimulated with 2 μg/ml FV peptide (FV GagL CTL epitope aa 85-93), P3C (1 μg/ml) in combination with anti-IL-6 (10 μg/ml) or not were added as indicated. The IFN-γ production by CD8^+^ T cells was measured by intracellular cytokine staining. Representative dot plots show the population of IFN-γ^+^ CD8^+^ T cells (lower panel). *(**B**)* Total splenocytes from FV-TCR TCR transgenic mice were stimulated with 2 μg/ml FV peptide (FV GagL CTL epitope aa 85-93), poly(I:C) (1 μg/ml) in combination with anti-IL-6 (10 μg/ml) or not were added as indicated. Supernatant was collected after 72 h and IFN-γ production was determined by ELISA. *(**C**)* Acute FV infected mice were treated twice with poly(I:C) at 4 dpi and 8 dpi. IL-6 blocking antibody was injected with poly(I:C) or not as indicated (n = 5 per group). Mice were sacrificed at 10 dpi and viable nucleated spleen cells were analyzed by flow cytometry. FV GagL specific CD8^+^ T cells were stained by MHC class I tetramers and analyzed for the effector cell marker CD43 expression. Cytokine production by splenic CD8^+^ T cells in response to restimulation was measured by intracellular cytokine staining. *(**D**)* The expression of granzyme B in CD43^+^ CD8 T cells was measured by intracellular staining (n = 5 per group). *(**E**)* Mice received the same treatment as in panel B and were sacrificed at 12 dpi (n = 8 per group). Viral loads in the spleen of infected mice were calculated by infectious center assay. ^*^p < 0.05; ^**^p < 0.01, ^***^p < 0.001.

To verify this hypothesis in an *in vitro* experiment, we utilized TCR transgenic CD8^+^ T cells specific for the DbGagL FV epitope (FV-TCR CD8^+^ T cells). Naive splenocytes from FV-TCR transgenic mice were stimulated with DbGagL FV peptide to induce proliferation and effector T cell differentiation. P3C was added to enhance T cell activation and anti-IL-6 or control antibody was included. Cells were restimulated with αCD3/αCD28 antibodies 3 days later. To also test a ligand for a different TLR poly(I:C), which signals through TRL3, was used. P3C stimulation significantly promoted the differentiation of naïve FV-TCR CD8^+^ T cells into IFN-γ–producing effector cells as measured by intracellular cytokine staining. Importantly, combining P3C stimulation and IL-6 blockade led to a further enhancement of effector CD8^+^ T cell differentiation. Compared to P3C stimulation alone, a more than 2-fold increase in the percentage or absolute numbers of IFN-γ–producing CD8^+^ T cells was detected when additional IL-6 blocking antibody was added (Fig. 6A). Similar results were obtained after poly(I:C) or CpG (Supplemental Fig. 5) was added to enhance T cell activation. Poly(I:C) stimulation significantly enhanced the IFN-γ production by FV-TCR CD8^+^ T cells but additional IL-6 blockade further augmented the IFN-γ production (Fig. 6B). These *in vitro* experiments indicate that IL-6 induction by different TLR ligands can result in counter-regulation of T cell activation.

To demonstrate the suppressive effect of TLR-induced IL-6 *in vivo* we performed an experiment with FV infected mice. We have previously shown that poly(I:C) promotes virus-specific CD8^+^ T cell responses in FV infection^23^, whereas P3C stimulation only moderately effected CD8^+^ T cells in FV infected mice (Supplemental Fig. 6). Thus, we used poly(I:C) for our *in vivo* experiments. FV infected mice received poly(I:C) treatment alone or in combination with an IL-6 blocking antibody at 4 and 8 dpi. As shown in our earlier study poly(I:C) treatment of FV infected mice alone did not enhance the numbers of virus-specific CD8^+^ T cells but augmented their effector functions in particular the production of cytokines (Fig. 6C). In contrast, additional IL-6 blockade resulted in a significant increase (over 60%) in absolute numbers of tetramer (specific for the immunodominant FV GagL epitope) positive CD8^+^ T cells (Fig. 6C). To analyze the effector function of these CD8^+^ T cells, we stained for cells producing the antiviral cytokines IFN-γ, IL-2 or TNF-α after *ex vivo* restimulation. IL-6 blockade further enhanced the ability of splenic CD8^+^ T cells to produce those cytokines compared to poly(I:C) treatment alone. Significant increases in absolute numbers of IFN-γ (100%), IL-2 (over 50%) and TNF-α (over 50%) producing CD8^+^ T cells were observed (Fig. 6C). Moreover, increased numbers of granzyme B producing CD43^+^ CD8^+^ T cells were observed in mice receiving additional IL-6 blocking antibody compared to mice treated with poly(I:C) alone (Fig. 6D).

It is well known that IL-6 plays an important role in CD4^+^ T cell differentiation^14^. Therefore, we examined the differentiation of CD4^+^ T cells in FV infected mice that received poly(I:C) and IL-6 blockade treatment. No significant difference in numbers of effector (CD44^+^ CD62L-) or memory (CD44^+^ CD62L^+^) CD4^+^ T cell was observed between poly(I:C) treated mice and mice receiving the combination therapy (Supplemental Fig. 7A). We also analyzed the expression of CD127 and KLRG1 on effector CD4^+^ T cells to evaluate whether the cells were terminal differentiated effector or memory cells. Again, no significant difference in numbers of terminally differentiated effector (CD127- KLRG1^+^) or memory (CD127^+^ KLRG1-) CD4^+^ T cells was observed between the two groups (Supplemental Fig. 7B). Therefore, it seems that CD4^+^ T cell differentiation was not affected by IL-6 blockade in poly(I:C) treated mice during FV infection.

To analyze the anti-viral effect of this augmented CD8+ T cell response we determined FV loads in the spleen of the treated mice. Compared to untreated controls, poly(I:C) treatment alone resulted in a very strong reduction (over 95%) in spleen viral loads and additional IL-6 blockade further reduced the already low viral loads by more than 60% (Fig. 6E). It is known that poly(I:C) can be recognized by the endosomal TLR3 and the cytoplasmic helicase MDA5. In our previous study^23^, we showed that both the TLR3 and the MDA5 pathway play a role in the antiviral effect of poly(I:C), but TLR-3 sensing clearly dominates this response. Therefore, our data indicate that IL-6 blockade enhances the antiviral effect of TLR-3 stimulation, but we cannot rule out that it may also effect RIG-I-like receptor mediated responses.

Taken together, TLR ligand induced IL-6 counter-regulates effector T cell responses *in vivo* and thus a combination therapy of TLR stimulation and IL-6 blockade may be an effective new approach for the therapy of infectious diseases.

## Discussion

Induction of robust virus-specific CD8^+^ T cell responses is regarded as an effective strategy not only to prevent but also to control chronic viral infections. Recently, TLR agonists have been considered as promising candidates for prophylactic and therapeutic immunotherapy in chronic viral infection because of their abilities to augment virus-specific CD8^+^ T cell responses^25^,^26^,^27^,^28^,^29^. In the present study, we investigated the role of TLR-induced IL-6 in regulating virus-specific CD8^+^ T cell responses. We show that IL-6 counter-regulates the promoting role of TLR agonist in stimulating virus-specific effector CD8^+^ T cell responses. This effect is mediated by a direct stimulation of CD8^+^ T cells by IL-6 which downregulates STAT4 phosphorylation and T-bet expression of CD8^+^ T cells. Moreover, combining TLR stimulation and IL-6 blockade results in enhanced virus-specific CD8^+^ T cell immunity *in vitro* and *in vivo*.

Since its successful cloning in 1986, IL-6 has been recognized as one of the most important inflammatory factors. For example, IL-6 has a profound effect on CD4 T cells survival and proliferation. IL-6 retains Bcl-2 expression of naive CD4 T cells and thus prolongs cell survival in *in vitro* T cell cultures^30^. It has also been shown that IL-6 protects CD4 T cells from activation induced cell death (AICD) by inhibiting Fas/FasL expression^31^,^32^. IL-6 enhances T cell proliferation during T cell activation and therefore it was described as a costimulatory molecule for T cell activation. Antigen specific CD4 T cells also expand more vigorously *in vivo* if exogenous IL-6 is provided during immunization. This is due to the ability of IL-6 to reduce the level of apoptosis among Ag-stimulated cells^16^. IL-6 also plays a critical role in the development of CD4^+^ but not CD8^+^ T cell memory to virus infections^33^. However, more and more evidence also reveals the anti-inflammatory properties of this pleiotropic cytokine. For instance, in a pancreatitis model it was shown that IL-6 deficiency leads to a more severe inflammatory response^34^. The absence of IL-6 also leads to enhanced levels of IFN-γ in response to systemic delivery of endotoxin into mice, and administration of recombinant IL-6 can abolish this effect^35^. Therefore, IL-6 should be defined as a factor that balances pro- and anti-inflammatory conditions and contributes to immune homeostasis.

Type 1 immunity relies on the differentiation of two major subsets of T lymphocytes, the CD4^+^ T helper (Th) cell and the CD8^+^ cytotoxic T lymphocytes (CTLs), whose direct inflammatory and cytotoxic responses are essential for the elimination of intracellular pathogens. In the last years, most studies on the influence of IL-6 on T cell functions have been focused on CD4^+^ T cells. It has become increasingly clear that IL-6 can shift the Th1/Th2 balance towards Th2 through two independent molecular mechanisms. First, IL-6 stimulation of CD4^+^ T cells leads to an upregulation of nuclear factor of activated T cells (NFAT), a transcription factor regulating IL-4 transcription, resulting in IL-4 expression and thereby Th2 polarization^36^,^37^. Second, IL-6 upregulates the expression of SOCS1 in CD4^+^ T cells, which inhibits IFN-γ signaling and Th1 differentiation^17^. For the first time, our current study provides evidence that IL-6 can also directly act on CD8^+^ T cells to suppress type 1 immunity. Different to the mechanisms observed in CD4^+^ T cells, we found that direct IL-6 stimulation on CD8^+^ T cells induces activation of the STAT3 signaling pathway and upregulation of SOCS3 but not enhanced SOCS1 expression. SOCS3 is known to inhibit IL-12-induced STAT4 activation^38^, which is important for the induction of T-bet expression in T cells^39^. Among the various signals involved in T cell activation, the transcription factor T-bet profoundly influences CD8^+^ T-cell differentiation into effector CTLs^29^. T-bet can regulate IFN-γ, perforin, and granzyme B transcription, and in its absence, CD8^+^ T cells fail to effectively differentiate from a naive T cell into an effector or memory T cell^40^,^41^. It has been demonstrated previously that T-cell specific overexpression of SOCS3 reduced expression of T-bet in T cells and protected from development of conA-induced hepatitis in mice^42^. These reports suggest the possibility that IL-6 may decrease T-bet expression in T cells through upregulation of SOCS3. In line with these reports, we observed a significant decrease of STAT4 activation and T-bet expression to be associated with reduced effector CD8^+^ T cell activation after IL-6 treatment. Thus, this observation suggests a new mechanism of regulating Th1/Th2 balance by IL-6.

IL-6 is one of the most common cytokines that APCs produce in response to TLR signaling during virus infections^43^. However, the precise roles of IL-6 in the protection from and/or the development of virus-induced disease remain unclear. Some studies suggest that IL-6 plays a protective role in viral infections^44^,^45^. In contrast, other studies suggested that viral infection induced vigorous IL-6 production may promote the disease development^46^. In addition, virus infected mice that produced high levels of IL-6 following administration of TLR ligands display exacerbated development of the disease^47^,^48^. Recent study in cytomegalovirus (CMV) patients revealed a correlation between serum IL-6 level with exhaustion of CMV-specific CD8^+^ T cells^49^. In Theiler’s murine encephalomyelitis virus (TMEV) infection, IL-6 and IL-17 synergistically promote viral persistence by inhibiting cytotoxic T cell function^50^. These contradictory reports indicate an ambiguous role of TLR-induced IL-6 on host immune responses during viral infections. Here, we show that TLR1/2 or TLR3 stimulation enhances virus-specific CD8^+^ T cell response in the FV infection model, but TLR-induced IL-6 counter-regulates this effect. In the absence of IL-6, the effector CD8^+^ T cell response was further amplified after TLR stimulation. Thus, our result adds evidence to the counter-regulative properties of TLR-induced IL-6 on inducing acquired immunity in viral infections. Similar to our finding, a recent report in tumor immunity demonstrated that inflammatory cytokines (such as IL-6) induced by TLR stimulation were not required for the induction of anti-tumor CTL responses^51^. Our current data implies that they might even be detrimental rather than beneficial.

In the past two decades, development and clinical implementation of TLR ligands have been topics of intense research. Targeted manipulation of TLR signaling has been applied clinically to boost vaccine effectiveness or promote a robust Th1-dominanated immune response against viral infection^52^. Therefore, strategies which improve the efficacy of TLR ligands in promoting adaptive immune responses bear a great value for clinical application. Here, we show that IL-6 blockade enhances the effect of TLR ligand therapy resulting in augmented virus-specific CD8^+^ T cell immunity. So far, several TLR ligands entered clinical trials for the treatment of chronic viral infections^52^. In 2010, an IL-6 signaling blocking antibody called “Tocilizumab” was approved by the U.S. Food and Drug Administration for the treatment of rheumatoid arthritis^20^. Thus, combining TLR ligand treatment and IL-6 signaling blockade may be a feasible new strategy for future clinical trials testing its efficacy in chronic infectious diseases.

Concerning the employment of IL-6 signaling blockade for the treatment of chronic viral diseases, the following questions remain: First, elevated levels of serum IL-6 have been found in many chronic viral infections, such as HBV, HCV and HIV^53^,^54^,^55^, which may contribute to the T cell dysfunction in these infections according to the mechanism we described in this study. Therefore, whether IL-6 signaling blockade alone or in combination with other therapeutic approaches may contribute to virus control needs to be addressed. Second, two types of IL-6 signal transduction mechanisms exist; the classic-signaling via membrane-bound IL-6 receptor (IL-6R) and the trans-signaling via soluble IL-6R^9^. It is believed that IL-6 trans-signaling is pro-inflammatory whereas classic IL-6 signaling is needed for the regenerative or anti-inflammatory activities of the cytokine. The IL-6 blocking antibody used in this study leads to a global inhibition of IL-6 signaling. However, whether a more selective blockade of IL-6 trans-signaling or the classic signaling results in better improvement of anti-viral CD8^+^ T cell responses remains unknown. These questions should be addressed in further studies.

In conclusion, these results have implications for our understanding of the role of TLR-induced pro-inflammatory cytokines in orchestrating T cell immunity as well as for the development of new strategies to overcome T cell dysfunction in chronic infectious diseases.

## Methods

### Mice

Inbred C57BL/6 wild-type mice, IL-6 knockout mice and DbGagL TCR tg (T cell receptor transgenic) mice were maintained under pathogen-free conditions. The DbGagL TCR tg mice were on a C57BL/6 or B6.SJL (CD45.1 congenic) background, and more than 90% of the CD8^+^ T cells contained a TCR specific for the DbGagL Friend Virus (FV) epitope (FV-TCR CD8^+^ T-cells)^21^. All mice were females, 8-10 weeks of age and were kept in the Animal Care Center, University of Duisburg-Essen, Essen, Germany. STAT3^loxP/loxP^ (STAT3 wild type) and STAT3^−/∆vav^ (STAT3 deficient) mice were kindly provided by Dr. Philipp A. Lang (Heinrich-Heine-University Dusseldorf, Dusseldorf, Germany). Experiments were conducted in accordance with the Guide for the Care and Use of Laboratory Animals and were reviewed and approved by the local Animal Care and Use Committee (Animal Care Center, University of Duisburg-Essen, Essen, Germany, and the district government of Düsseldorf, Germany).

### Reagents and antibodies

Agonists for TLR1/2 (palmitoyl-3-cysteine-serinelysine-4, P3C) and TLR3 (polyinosine-polycytidylic acid, poly I:C) were purchased from InvivoGen (San Diego, CA). Recombinant mouse IL-6 was purchased from eBioscience (San Diego, CA). Blocking antibody anti-IL-6 was purchased from BioXCell (Lebanon, NH).

### Cell isolation

Isolation of LSECs, splenic dendritic cells (DCs) and CD8^+^ T cells was performed as described previously^7^. The purity of the cell fractions was monitored by flow cytometry and was greater than 98% in all cases. All cell fractions contained less than 5% dead cells after the separation procedure.

### Flow cytometry

Cell-surface and intracellular staining for flow cytometry analysis was performed using BD Biosciences (Heidelberg, Germany) or eBioscience (Frankfurt, Germany) reagents. Intracellular granzyme B and IFN-γ staining was performed as described^7^,^56^. Data were acquired using a LSRII flow cytometer (BD Biosciences, Heidelberg, Germany) and analyzed using FlowJo software (Tree Star, Inc., Ashland, Oregon). Cell debris and dead cells were excluded from the analysis based on scatter signals and 7-Amino-actinomycin D (7-AAD) or Fixable Viability Dye eFluor® 780 fluorescence.

### T cell activation assay

Red blood cell-depleted splenocytes were cultured at 4 × 10^5^ cells/well in a total volume of 200 μl. Splenocytes were stimulated with 1 μg/ml anti-CD3 and 1 μg/ml anti-CD28. Cell-free supernatants were collected, and subjected to assays to measure IFN-γ production using cytokine ELISA kits (eBioscience, Frankfurt, Germany).

### Analysis of CD8^+^ T cell function

Purified FV TCR tg CD8^+^ T cells were coincubated with 2 μg/ml FV peptide (FV GagL CTL epitope aa 85–93) loaded APCs or purified wild type CD8^+^ T cells were cocultured with CD3/CD28 Dynabeads for 3 days. Prior to intracellular cytokine staining, CD8^+^ T cells were restimulated by 10 μg/ml of anti-CD3 antibody and 1 μg/ml of anti-CD28 antibody (BD Pharmingen, Heidelberg, Germany) for 5 h in the presence of 5 μg/ml of brefeldin A (Sigma-Aldrich) and analyzed for activation by flow cytometry. The ratio of CD8^+^ T cells to APCs was 2:1.

### *In vitro* cytotoxicity assays

The *in vitro* cytotoxicity assays were performed as described earlier^57^. Briefly, splenocytes were either loaded with FV peptide and labeled CFSE^high^ (2 μmol/L), or remained unloaded labeled CFSE^low^ (0.2 μmol/L) as controls. CFSE labeled target cells and effector T cells were mixed at indicated ratios and specific killing of 5 × 10^3^ target cells *in vitro* was determined after 4 hours. Specific kill was determined using the following formula: % Specific Kill = 100−(100*[CFSE^high^/CFSE^low^]sample/[CFSE^high^/CFSE^low^]control).

### Quantitative reverse transcription–polymerase chain reaction (RT-PCR)

Total RNA was isolated from 1 × 10^6^ to 10 × 10^6^ cells using Trizol (Gibco). One-step real time RT-PCR was carried out with the QuantiTect SYBR Green RT-PCR Kit (Qiagen, Hilden, Germany) on the iCycler real-time amplification system (Bio-Rad, Hercules, CA) as described previously^58^.

### Friend virus infection

Acute Friend virus infection was performed as described previously^59^. All mice were sacrificed and spleens were taken at 10 days post infection (dpi) for T cell function analysis or at 12 dpi for determination of viral titers.

### Infectious center assays

Analyses of Friend virus loads were performed as described previously^7^,^60^. Briefly, , single cell suspensions from spleens of infected mice were prepared, plated onto susceptible Mus dunni cells^61^, and cocultivated for 3 days. The cells were fixed with ethanol and stained with F-MuLV envelope-specific mAb 720^62^. The infectious centers were visualized by using peroxidase-conjugated goat anti-mouse antibodies and AEC for the detection of foci.

### Statistical Analysis

Statistical analyses were performed using GraphPad Prism software version 5 (GraphPad Software Inc., San Diego, CA). Statistical comparison of two groups were done using the Mann-Whitney test and the unpaired t test. Statistics comparing more than two groups was done using the Kruskal-Wallis one-way analysis of variance on ranks and the Dunns multiple comparison procedure.

## Author Contributions

J.L. wrote the main manuscript text and prepared figures; W.W., K.D., M.T. and J.L. performed the experiments and analyzed the data; K.G., K.L., H.Y., J.W., D.Y., M.L., M.R. and U.D. provided experimental material and made critical revision for the manuscript. All authors reviewed the manuscript.

## Additional Information

**How to cite this article**: Wu, W. *et al*. TLR ligand induced IL-6 counter-regulates the anti-viral CD8+ T cell response during an acute retrovirus infection. *Sci. Rep.* **5**, 10501; doi: 10.1038/srep10501 (2015).

## References

[b1] Akira S. & Takeda K. Toll-like receptor signalling. Nat Rev Immunol 4, 499–511 (2004). 15229469 10.1038/nri1391

[b2] Iwasaki A. & Medzhitov R. Toll-like receptor control of the adaptive immune responses. Nat Immunol 5, 987–995 (2004). 15454922 10.1038/ni1112

[b3] Reise Sousa C. Toll-like receptors and dendritic cells: for whom the bug tolls. Semin Immunol 16, 27–34 (2004). 14751761 10.1016/j.smim.2003.10.004

[b4] Banchereau J. & Steinman R. M. Dendritic cells and the control of immunity. Nature 392, 245–252 (1998). 9521319 10.1038/32588

[b5] Janeway C. A. Jr. & Medzhitov R. Innate immune recognition. Annu Rev Immunol 20, 197–216 (2002). 11861602 10.1146/annurev.immunol.20.083001.084359

[b6] Trinchieri G. Interleukin-12 and the regulation of innate resistance and adaptive immunity. Nat Rev Immunol 3, 133–146 (2003). 12563297 10.1038/nri1001

[b7] Liu J. *et al.* TLR1/2 ligand-stimulated mouse liver endothelial cells secrete IL-12 and trigger CD8+ T cell immunity *in vitro*. J Immunol 191, 6178–6190 (2013). 24227786 10.4049/jimmunol.1301262

[b8] Scheller J., Chalaris A., Schmidt-Arras D. & Rose-John S. The pro- and anti-inflammatory properties of the cytokine interleukin-6. Biochim Biophys Acta 1813, 878–888 (2011). 21296109 10.1016/j.bbamcr.2011.01.034

[b9] Jones S. A. Directing transition from innate to acquired immunity: defining a role for IL-6. J Immunol 175, 3463–3468 (2005). 16148087 10.4049/jimmunol.175.6.3463

[b10] Strestik B. D., Olbrich A. R., Hasenkrug K. J. & Dittmer U. The role of IL-5, IL-6 and IL-10 in primary and vaccine-primed immune responses to infection with Friend retrovirus (Murine leukaemia virus). J Gen Virol 82, 1349–1354 (2001). 11369878 10.1099/0022-1317-82-6-1349

[b11] Lauder S. N. *et al.* Interleukin-6 limits influenza-induced inflammation and protects against fatal lung pathology. Eur J Immunol 43, 2613–2625 (2013). 23857287 10.1002/eji.201243018 PMC3886386

[b12] Hoge J. *et al.* IL-6 controls the innate immune response against Listeria monocytogenes via classical IL-6 signaling. J Immunol 190, 703–711 (2013). 23241882 10.4049/jimmunol.1201044

[b13] Kishimoto T. The biology of interleukin-6. Blood 74, 1–10 (1989). 2473791

[b14] Dienz O. & Rincon M. The effects of IL-6 on CD4 T cell responses. Clin Immunol 130, 27–33 (2009). 18845487 10.1016/j.clim.2008.08.018 PMC2660866

[b15] Dodge I. L., Carr M. W., Cernadas M. & Brenner M. B. IL-6 production by pulmonary dendritic cells impedes Th1 immune responses. J Immunol 170, 4457–4464 (2003). 12707321 10.4049/jimmunol.170.9.4457

[b16] Rochman I., Paul W. E. & Ben-Sasson S. Z. IL-6 increases primed cell expansion and survival. J Immunol 174, 4761–4767 (2005). 15814701 10.4049/jimmunol.174.8.4761

[b17] Diehl S. *et al.* Inhibition of Th1 differentiation by IL-6 is mediated by SOCS1. Immunity 13, 805–815 (2000). 11163196 10.1016/s1074-7613(00)00078-9

[b18] Korn T., Bettelli E., Oukka M. & Kuchroo V. K. IL-17 and Th17 Cells. Annu Rev Immunol 27, 485–517 (2009). 19132915 10.1146/annurev.immunol.021908.132710

[b19] Bettelli E. *et al.* Reciprocal developmental pathways for the generation of pathogenic effector TH17 and regulatory T cells. Nature 441, 235–238 (2006). 16648838 10.1038/nature04753

[b20] Tanaka T. & Kishimoto T. The biology and medical implications of interleukin-6. Cancer Immunol Res 2, 288–294 (2014). 24764575 10.1158/2326-6066.CIR-14-0022

[b21] Dittmer U. *et al.* Functional impairment of CD8(+) T cells by regulatory T cells during persistent retroviral infection. Immunity 20, 293–303 (2004). 15030773 10.1016/s1074-7613(04)00054-8

[b22] Olbrich A. R. *et al.* Effective postexposure treatment of retrovirus-induced disease with immunostimulatory DNA containing CpG motifs. J Virol 76, 11397–11404 (2002). 12388700 10.1128/JVI.76.22.11397-11404.2002 PMC136771

[b23] Gibbert K. *et al.* Polyinosinic-polycytidylic acid treatment of Friend retrovirus-infected mice improves functional properties of virus-specific T cells and prevents virus-induced disease. J Immunol 185, 6179–6189 (2010). 20943997 10.4049/jimmunol.1000858

[b24] Yoshimura A., Suzuki M., Sakaguchi R., Hanada T. & Yasukawa H. SOCS, Inflammation, and Autoimmunity. Front Immunol 3, 20 (2012). 22566904 10.3389/fimmu.2012.00020 PMC3342034

[b25] Komai-Koma M., Jones L., Ogg G. S., Xu D. & Liew F. Y. TLR2 is expressed on activated T cells as a costimulatory receptor. Proc Natl Acad Sci USA 101, 3029–3034 (2004). 14981245 10.1073/pnas.0400171101 PMC365739

[b26] Cottalorda A. *et al.* TLR2 engagement on CD8 T cells lowers the threshold for optimal antigen-induced T cell activation. Eur J Immunol 36, 1684–1693 (2006). 16761317 10.1002/eji.200636181

[b27] Vitiello A. *et al.* Development of a lipopeptide-based therapeutic vaccine to treat chronic HBV infection. I. Induction of a primary cytotoxic T lymphocyte response in humans. J Clin Invest 95, 341–349 (1995). 7814635 10.1172/JCI117662 PMC295437

[b28] Jackson D. C. *et al.* A totally synthetic vaccine of generic structure that targets Toll-like receptor 2 on dendritic cells and promotes antibody or cytotoxic T cell responses. Proc Natl Acad Sci USA 101, 15440–15445 (2004). 15489266 10.1073/pnas.0406740101 PMC523460

[b29] Geng D. *et al.* When Toll-like receptor and T-cell receptor signals collide: a mechanism for enhanced CD8 T-cell effector function. Blood 116, 3494–3504 (2010). 20696947 10.1182/blood-2010-02-268169 PMC2981476

[b30] Teague T. K., Marrack P., Kappler J. W. & Vella A. T. IL-6 rescues resting mouse T cells from apoptosis. J Immunol 158, 5791–5796 (1997). 9190930

[b31] Ayroldi E. *et al.* Interleukin-6 (IL-6) prevents activation-induced cell death: IL-2-independent inhibition of Fas/fasL expression and cell death. Blood 92, 4212–4219 (1998). 9834226

[b32] Kishimoto H. & Sprent J. Strong TCR ligation without costimulation causes rapid onset of Fas-dependent apoptosis of naive murine CD4+ T cells. J Immunol 163, 1817–1826 (1999). 10438914

[b33] Longhi M. P. *et al.* Interleukin-6 is crucial for recall of influenza-specific memory CD4 T cells. PLoS Pathog 4, e1000006 (2008). 18389078 10.1371/journal.ppat.1000006 PMC2279258

[b34] Cuzzocrea S. *et al.* Absence of endogenous interleukin-6 enhances the inflammatory response during acute pancreatitis induced by cerulein in mice. Cytokine 18, 274–285 (2002). 12161103 10.1006/cyto.2002.0883

[b35] Xing Z. *et al.* IL-6 is an antiinflammatory cytokine required for controlling local or systemic acute inflammatory responses. J Clin Invest 101, 311–320 (1998). 9435302 10.1172/JCI1368 PMC508569

[b36] Murphy K. M. *et al.* Signaling and transcription in T helper development. Annu Rev Immunol 18, 451–494 (2000). 10837066 10.1146/annurev.immunol.18.1.451

[b37] Diehl S. *et al.* Induction of NFATc2 expression by interleukin 6 promotes T helper type 2 differentiation. J Exp Med 196, 39–49 (2002). 12093869 10.1084/jem.20020026 PMC2194007

[b38] Yamamoto K., Yamaguchi M., Miyasaka N. & Miura O. SOCS-3 inhibits IL-12-induced STAT4 activation by binding through its SH2 domain to the STAT4 docking site in the IL-12 receptor beta2 subunit. Biochem Biophys Res Commun 310, 1188–1193 (2003). 14559241 10.1016/j.bbrc.2003.09.140

[b39] Yang Y., Ochando J. C., Bromberg J. S. & Ding Y. Identification of a distant T-bet enhancer responsive to IL-12/Stat4 and IFNgamma/Stat1 signals. Blood 110, 2494–2500 (2007). 17575072 10.1182/blood-2006-11-058271 PMC1988915

[b40] Intlekofer A. M. *et al.* Effector and memory CD8+ T cell fate coupled by T-bet and eomesodermin. Nat Immunol 6, 1236–1244 (2005). 16273099 10.1038/ni1268

[b41] Sullivan B. M., Juedes A., Szabo S. J., von Herrath M. & Glimcher L. H. Antigen-driven effector CD8 T cell function regulated by T-bet. Proc Natl Acad Sci USA 100, 15818–15823 (2003). 14673093 10.1073/pnas.2636938100 PMC307651

[b42] Fushimi S. *et al.* Forced expression of suppressor of cytokine signaling 3 in T cells protects the development of concanavalin A-induced hepatitis in mice. Clin Immunol 133, 437–446 (2009). 19766538 10.1016/j.clim.2009.08.015

[b43] Akira S., Uematsu S. & Takeuchi O. Pathogen recognition and innate immunity. Cell 124, 783–801 (2006). 16497588 10.1016/j.cell.2006.02.015

[b44] Rodriguez M., Pavelko K. D., McKinney C. W. & Leibowitz J. L. Recombinant human IL-6 suppresses demyelination in a viral model of multiple sclerosis. J Immunol 153, 3811–3821 (1994). 7930598

[b45] Hosel M. *et al.* Not interferon, but interleukin-6 controls early gene expression in hepatitis B virus infection. Hepatology 50, 1773–1782 (2009). 19937696 10.1002/hep.23226

[b46] Hou W., Kang H. S. & Kim B. S. Th17 cells enhance viral persistence and inhibit T cell cytotoxicity in a model of chronic virus infection. J Exp Med 206, 313–328 (2009). 19204109 10.1084/jem.20082030 PMC2646583

[b47] Jin Y. H. *et al.* TLR3 signaling is either protective or pathogenic for the development of Theiler’s virus-induced demyelinating disease depending on the time of viral infection. J Neuroinflammation 8, 178 (2011). 22189096 10.1186/1742-2094-8-178 PMC3293102

[b48] Pullen L. C., Park S. H., Miller S. D., Dal Canto M. C. & Kim B. S. Treatment with bacterial LPS renders genetically resistant C57BL/6 mice susceptible to Theiler’s virus-induced demyelinating disease. J Immunol 155, 4497–4503 (1995). 7594613

[b49] Kato T. *et al.* Correlations of programmed death 1 expression and serum IL-6 level with exhaustion of cytomegalovirus-specific T cells after allogeneic hematopoietic stem cell transplantation. Cell Immunol 288, 53–59 (2014). 24657340 10.1016/j.cellimm.2014.02.007

[b50] Hou W., Jin Y. H., Kang H. S. & Kim B. S. Interleukin-6 (IL-6) and IL-17 synergistically promote viral persistence by inhibiting cellular apoptosis and cytotoxic T cell function. J Virol 88, 8479–8489 (2014). 24829345 10.1128/JVI.00724-14 PMC4135960

[b51] Matsumoto M. *et al.* Defined TLR3-specific adjuvant that induces NK and CTL activation without significant cytokine production *in vivo*. Nat Commun 6, 6280 (2015). 25692975 10.1038/ncomms7280

[b52] Hedayat M., Netea M. G. & Rezaei N. Targeting of Toll-like receptors: a decade of progress in combating infectious diseases. Lancet Infect Dis 11, 702–712 (2011). 21719349 10.1016/S1473-3099(11)70099-8

[b53] Ueyama M. *et al.* Serum interleukin-6 levels correlate with resistance to treatment of chronic hepatitis C infection with pegylated-interferon-alpha2b plus ribavirin. Antivir Ther 16, 1081–1091 (2011). 22024524 10.3851/IMP1864

[b54] Breen E. C. *et al.* Infection with HIV is associated with elevated IL-6 levels and production. J Immunol 144, 480–484 (1990). 2295799

[b55] Zhang F. *et al.* Roles of circulating soluble interleukin (IL)-6 receptor and IL-6 receptor expression on CD4+ T cells in patients with chronic hepatitis B. Int J Infect Dis 15, e267–271 (2011). 21295508 10.1016/j.ijid.2010.12.008

[b56] Zelinskyy G., Kraft A. R., Schimmer S., Arndt T. & Dittmer U. Kinetics of CD8+ effector T cell responses and induced CD4+ regulatory T cell responses during Friend retrovirus infection. Eur J Immunol 36, 2658–2670 (2006). 16981182 10.1002/eji.200636059

[b57] Kern M. *et al.* Virally infected mouse liver endothelial cells trigger CD8+ T-cell immunity. Gastroenterology 138, 336–346 (2010). 19737567 10.1053/j.gastro.2009.08.057

[b58] Liu J. *et al.* A new splice variant of the major subunit of human asialoglycoprotein receptor encodes a secreted form in hepatocytes. PLoS One 5, e12934 (2010). 20886072 10.1371/journal.pone.0012934 PMC2944864

[b59] Hasenkrug K. J., Brooks D. M. & Dittmer U. Critical role for CD4(+) T cells in controlling retrovirus replication and spread in persistently infected mice. J Virol 72, 6559–6564 (1998). 9658100 10.1128/jvi.72.8.6559-6564.1998 PMC109830

[b60] Dittmer U., Brooks D. M. & Hasenkrug K. J. Characterization of a live-attenuated retroviral vaccine demonstrates protection via immune mechanisms. J Virol 72, 6554–6558 (1998). 9658099 10.1128/jvi.72.8.6554-6558.1998 PMC109828

[b61] Lander M. R. & Chattopadhyay S. K. A Mus dunni cell line that lacks sequences closely related to endogenous murine leukemia viruses and can be infected by ectropic, amphotropic, xenotropic, and mink cell focus-forming viruses. J Virol 52, 695–698 (1984). 6092693 10.1128/jvi.52.2.695-698.1984 PMC254577

[b62] Robertson M. N. *et al.* Production of monoclonal antibodies reactive with a denatured form of the Friend murine leukemia virus gp70 envelope protein: use in a focal infectivity assay, immunohistochemical studies, electron microscopy and western blotting. J Virol Methods 34, 255–271 (1991). 1744218 10.1016/0166-0934(91)90105-9

